# Sex Differences in Neural Responses to the Perception of Social Interactions

**DOI:** 10.3389/fnhum.2020.565132

**Published:** 2020-09-11

**Authors:** Guangfei Li, Yu Chen, Wuyi Wang, Isha Dhingra, Simon Zhornitsky, Xiaoying Tang, Chiang-Shan R. Li

**Affiliations:** ^1^Department of Biomedical Engineering, School of Life Sciences, Beijing Institute of Technology, Beijing, China; ^2^Department of Psychiatry, Yale University School of Medicine, New Haven, CT, United States; ^3^Department of Neuroscience, Yale University School of Medicine, New Haven, CT, United States; ^4^Interdepartmental Neuroscience Program, Yale University School of Medicine, New Haven, CT, United States

**Keywords:** social emotion, social interaction, gender, HCP, fMRI

## Abstract

Social interaction is critical to emotional well-being. Previous studies have suggested sex differences in the perception of social interaction. However, the findings depend on the nature of interactions and whether it involves facial emotions. Here, we explored sex differences in neural responses to the perception of social interaction using the Human Connectome Project data. Participants (*n* = 969, 505 women) were engaged in a social cognition task with geometric objects moving and colliding to simulate social interaction. Behaviorally, men relative to women demonstrated higher accuracy in perceiving social vs. random interactions. Men vs. women showed higher activation in the right superior temporal gyrus, bilateral occipital and posterior cingulate cortex and precuneus, and women vs. men showed higher activation in the right inferior frontal cortex, during exposure to social vs. random interactions. In whole-brain regressions, the differences in accuracy rate in identifying social vs. random interactions (AR_*SOC*_ – AR_*RAN*_) were associated with higher activation in the paracentral lobule (PCL) and lower activation in bilateral anterior insula (AI), pre-supplementary motor area (preSMA), and left middle frontal gyrus (MFG) in men and women combined, lower activation in bilateral AI, preSMA and left MFG in men alone, and higher activation in the PCL and the medial orbitofrontal cortex in women alone. The latter sex differences were confirmed by slope tests. Further, the PCL activity mediated the correlation between an internalizing syndromal score, as assessed by the Achenbach Self-Report, and (AR_*SOC*_ – AR_*RAN*_) across all subjects. These findings highlighted sex differences in the behavioral and neural processes underlying the perception of social interaction, as well as the influence of internalizing traits on these processes.

## Introduction

### Sex Differences in the Perception of Social Interaction

Perception of social interaction is central to “theory of mind” processing and inter-personal engagements. Sex differences in the perception of verbal (Lee et al., 2017) or non-verbal (Hall, 1978; Rasmussen and Jiang, 2019) social interaction may reflect a consequence of evolutionary pressures on reproduction and survival (Buss, 1995; Wood and Eagly, 2002). In most mammals, as the primary caretakers of the offspring, females relative to males exhibited superiority in perceiving social interactions, conferring an advantage in recognizing and responding to infants’ needs (Babchuk et al., 1985; Maestripieri and Pelka, 2002; Simpson et al., 2016). In humans, a magnetoencephalography study of healthy adults showed gamma neuromagnetic response peaking earlier in females than in males during animated social interaction, suggesting that women may anticipate social interaction by predicting others’ actions, whereas men require accumulation of more sensory evidence for social decisions (Pavlova et al., 2010). Furthermore, the sex differences appear to manifest early in life. For instance, in macaque monkeys, female relative to male infants looked more frequently at conspecifics’ faces at 2–3 weeks, and exhibited more affiliative behaviors, including gesturing and looking at human caretakers, at 4–5 weeks (Simpson et al., 2016).

However, females are not always more proficient in understanding social signals, and their social abilities may be particularly affected by illnesses (Pavlova, 2017). Additionally, earlier reports of sex differences in the sensitivity to social interaction could largely be accounted for by higher female sensitivity to human faces and facial emotions (Fischer et al., 2018). Although women were better at recognizing emotions and express themselves more easily, men showed greater responses to threatening social cues (Kret and De Gelder, 2012). Together, these findings suggest that sex differences in the perception of social interaction may vary according to the nature of social stimuli.

### Imaging Perception of Social Interaction

The neural processes supporting the perception of social interaction, typically delivered via visual stimuli, are widely distributed in the frontal, parietal, and temporal areas (Kujala et al., 2012; Deuse et al., 2016; Powers et al., 2016; Sapey-Triomphe et al., 2017). Higher activation has been found in the medial prefrontal cortex (mPFC), inferior frontal gyrus (IFG), and superior temporal gyrus (STG) in the perception of social vs. non-social interaction (Centelles et al., 2011). The activation of the mPFC was associated with active engagement in social interaction while the precuneus responded to passive observation of social interaction (Schilbach et al., 2006). The posterior STG was not only sensitive to the presence of social interaction (vs. independent action) but also to the content of qualitatively different interactions (i.e., competition vs. cooperation), suggesting its role in evaluating social interactions (Walbrin et al., 2018). Cooperative (e.g., helping each other climb a tree) and affective (e.g., shaking hands) interactions both activated a bilateral network, extending from the fusiform gyrus to posterior middle/superior temporal and posterior parietal cortices (Arioli et al., 2018). In addition, perceiving another person’s pain led to regional activities involved in social interaction and emotion regulation, including those in the temporo-parietal junction, mPFC, and IFG (Akitsuki and Decety, 2009). The examination of the neural processes underlying the perception of social interaction has advanced the understanding of social cognition. On the other hand, no fMRI studies to our knowledge have investigated sex differences in these regional responses.

### A Potential Role of Internalizing Personality Trait

Sex differences in the perception of social interaction may be influenced by individual differences in internalizing personality trait. People with higher avoidant trait are particularly sensitive to social interactions (Eggum et al., 2009). Those with avoidant personality disorder consider themselves unappealing or socially inadequate, and fear coping with social interactions (APA, 2013). Children with higher internalizing traits were less competent socially and exhibited more significant anxiety during social interactions (Bornstein et al., 2010). In self-reported personality measures of young adults, men showed a slight trend to endorse more criteria than women for avoidant personality disorder (Klonsky et al., 2002). Thus, individual differences in internalizing traits may directly impact the perception of social interaction. It is important to investigate whether individuals varying in internalizing traits show differences in the sensitivity to perceiving social interactions, and whether/how the neural processes underlying the perception of social interaction vary with the individual differences.

### The Present Study

We addressed these gaps in research by taking advantage of a large imaging data set collected of the Human Connectome Project (HCP). The imaging paradigm of the HCP social task comprised blocks of geometric stimuli simulating social interactions, and others simulating random interactions. Participants were required to indicate whether they perceived social or random interaction and the accuracy rates were quantified for each block. We examined whether the differences in accuracy rate were sex-dependent and related to individual differences in an internalizing trait. We contrasted the brain activation in social and random blocks (brain activation in social block minus brain activation in random block) to identify regional responses to the perception of social interaction, and compared men and women in these regional processes. Further, we used the differences in accuracy rate and in reaction time as a regressor for whole-brain regressions in men and women combined as well as separately to examine the neural correlates of individual ability in identifying social interactions. For the neural correlates identified specifically of men or of women, we extracted the β estimates and confirmed or refuted the sex difference with a slope test. Our goal was to reveal how men and women may identify visual stimuli simulating social interactions differently and the neural processes underlying the sex differences. These findings may contribute to the research of social emotional processing in neuropsychiatric conditions (e.g., autism spectrum disorder) known to show sex biases in incidence.

## Materials and Methods

### Dataset and Assessment of Social Emotions

For the present study, we have obtained permission from the HCP to use both the Open and Restricted Access data. As in our previous work (Li et al., 2020), we employed the 1200 Subjects Release (S1200) data set, including behavioral and 3T MR imaging data of 1206 healthy young adult participants (1113 with structural MR scans) collected from 2012 to 2015, for this study. The data of a total of 969 adults (505 women; age = 29.5 ± 3.5 years) were obtained from the HCP (Table 1). All subjects were physically healthy with no severe neurodevelopmental, neuropsychiatric, or neurological disorders. Subject recruitment procedures and informed consents, including consent to share de-identified data, were approved by the Washington University Institutional Review Board.

**TABLE 1:** Demographic, clinical, and behavioral measures.

	Men (*n* = 464)	Women (*n* = 505)	*p-*Value*	*t*_*dof*_
Age, years	27.9 ± 3.6	29.5 ± 3.5	0.000	*t*_967_ = −7.083
Education, years	14.8 ± 1.9	15.0 ± 1.9	0.084	*t*_967_ = −1.731
ASR Intn Score	10.6 ± 9.2	10.6 ± 9.1	0.502	*t*_965_ = −0.057
AR_*SOC*_, %	95.6 ± 11.5	93.3 ± 13.0	0.001	*t*_955_ = 2.832
AR_*RAN*_, %	85.4 ± 19.0	86.2 ± 19.1	0.567	*t*_955_ = −0.640
AR_*SOC*_ – AR_*RAN*_, %	10.2 ± 20.6	7.2 ± 22.3	0.020	*t*_955_ = 2.181
RT_*SOC*_, ms	1073 ± 306	1069 ± 309	0.833	*t*_955_ = 0.223
RT_*RAN*_, ms	1062 ± 331	1013 ± 300	0.007	*t*_955_ = 2.379
RT_*SOC*_ – RT_*RAN*_, ms	11 ± 342	56 ± 333	0.019	*t*_955_ = −2.033

All values are mean ± SD. *Two-sample *t*-test (for age and years of education) or covariance analysis with age and years of education as covariates (other variables). dof: degree of freedom; ASR Intn Score: ASR Internalizing score; AR_*SOC*_/AR_*RAN*_: the accuracy rate of social/random blocks; RT_*SOC*_/RT_*RAN*_: the reaction time of social/random blocks. A total of 10 subjects (4 women) were short of RT and/or AR in the raw data of HCP; as a result, 959 subjects (501 women) were included in the RT and AR statistics. ASR: Achenbach Adult Self Report.

All participants were assessed with the Achenbach Adult Self-Report or ASR (Achenbach, 2009) Syndrome Scales. There were a total of 120 items in 12 subscales (item for example): anxious/depressed (‘I feel lonely’), withdrawn (‘I don’t get along with other people’), somatic complaints (‘I feel dizzy or lightheaded’), thought problems (‘I can’t get my mind off certain thoughts’), attention problems (‘I am too forgetful’), aggressive behavior (‘I argue a lot’), rule-breaking behavior (‘I break rules at work or elsewhere’), intrusive behavior (‘I try to get a lot of attention’), other problems (‘I have trouble sitting still’), as well as all critical items (items of general clinical concern), an internalizing subscale (consisting of anxious/depressed, withdrawn and somatic complaints), and an externalizing subscale (consisting of aggressive, rule-breaking, and Intrusive behavior). Participants were required to circle 0 (not true), 1 (somewhat or sometimes true), or 2 (very or often true) for each item to describe themselves over the past 6 months.

### Behavioral Task for fMRI and Behavioral Data Analysis

Each participant completed two runs of a social cognition task each with five blocks. The task showed robust activation in brain regions associated with social cognition (Castelli et al., 2000; Wheatley et al., 2007). The first run contained two social and three random blocks in a fixed order: social – random – random – social – random; the second run contained three social and two random blocks in a fixed order: social – social – random – social – random. Developed by Castelli et al. (2000) and Wheatley et al. (2007), video clips [20 s, shortened from the original 40-s versions (Barch et al., 2013)] of geometric objects (triangles, squares, circles) “interacted” in some way to simulate causal actions in social blocks, and moved randomly in random blocks. At the end of each video clip, subjects were allowed 3 s to indicate whether the objects interacted socially (as if the objects took into account each other’s feelings and thoughts), not sure, or no interaction (i.e., movement appearing random). Of all HCP subjects, 158 did not participate or participate fully in the social cognition task. Further, 79 subjects who had head movements greater than 2 mm in translation or 2 degrees in rotation or for whom the images failed in registration to the template were excluded. As a result, a total of 969 (505 women) subjects were included in the current study. Ten subjects’ RT and/or accuracy rate were missing from the data, and thus 959 (501 women) were included in the analyses of RT and accuracy rate (AR) as well as in the imaging data analyses on the basis of RT and AR.

For both AR and RT, we performed an analysis of variance (ANOVA) with sex (men vs. women) as a between-subject variable and block type (social vs. random) as a within-subject variable, with age and years of education as covariates.

We evaluated whether the difference in AR and in RT between the social and random blocks – (AR_*SOC*_ – AR_*RAN*_); (RT_*SOC*_ – RT_*RAN*_) – was correlated with each of the 12 Achenbach Adult Self-Report Syndrome Scale subscores (Achenbach, 2009), as described above, with age, sex, years of education as covariates across all subjects and with age and years of education as covariates in men and women separately.

### Imaging Protocol and Data Preprocessing

MRI was done using a customized 3 T Siemens Connectome Skyra with a standard 32-channel Siemens receiver head coil and a body transmission coil. T1-weighted high-resolution structural images were acquired using a 3D MPRAGE sequence with 0.7 mm isotropic resolution (FOV = 224 × 224 mm, matrix = 320 × 320, 256 sagittal slices, TR = 2400 ms, TE = 2.14 ms, TI = 1000 ms, FA = 8°) and used to register functional MRI data to a standard brain space. FMRI data were collected using gradient-echo echo-planar imaging (EPI) with 2.0 mm isotropic resolution (FOV = 208 × 180 mm, matrix = 104 × 90, 72 slices, TR = 720 ms, TE = 33.1 ms, FA = 52°, multi-band factor = 8, 274 frames, ∼ 3 m and 27 s/run).

Imaging data were analyzed with Statistical Parametric Mapping (SPM8, Welcome Department of Imaging Neuroscience, University College London, United Kingdom), following our published routines (Wang et al., 2019; Zhang et al., 2019; Zhornitsky et al., 2019b). Images of each individual subject were first realigned (motion corrected). A mean functional image volume was constructed for each subject per run from the realigned image volumes. These mean images were co-registered with the high-resolution structural MPRAGE image and then segmented for normalization with affine registration followed by non-linear transformation. The normalization parameters determined for the structural volume were then applied to the corresponding functional image volumes for each subject. Finally, the images were smoothed with a Gaussian kernel of 4 mm at Full Width at Half Maximum.

### Imaging Data Modeling and Statistics

We modeled the BOLD signals to identify regional brain responses to social vs. random block through the contrast [social - random]. A statistical analytical block design was constructed for each individual subject, using a general linear model (GLM) with block onsets of social or random blocks convolved with a canonical hemodynamic response function. Realignment parameters in all six dimensions were entered in the model as covariates. Serial autocorrelation caused by aliased cardiovascular and respiratory effects was corrected by a first-degree autoregressive model. The GLM estimated the component of variance that could be explained by each of the regressors.

In the first-level analysis, we constructed for individual subjects a contrast of social vs. random block to evaluate brain regions that responded differently during viewing of social vs. random video clips. The contrast images (difference in β) of the first-level analysis were then used for the second-level group statistics.

In group analyses, we conducted a one-sample *t*-test to identify regional responses to social vs. random stimuli for men and women together and separately. We compared men and women in a two-sample *t*-test of the same contrast with age and years of education as covariates to evaluate sex differences in regional responses. To examine how regional brain responses to the contrast varied with individual differences in AR, we conducted a whole-brain multiple regression on social vs. random against “AR_*SOC*_ – AR_*RAN*_” and “RT_*SOC*_ – RT_*RAN*_” in men and women together, with age, sex and years of education as covariates, and in men and women separately, with age and years of education as covariates. Following current reporting standards, all imaging results were evaluated with voxel *p* < 0.001, uncorrected, in combination with a cluster *p* < 0.05, corrected for family-wise error (FWE) of multiple comparisons, on the basis of Gaussian random field theory, as implemented in SPM (Poldrack et al., 2008). All voxel activations were reported in Montreal Neurological Institute (MNI) coordinates.

In ROI analysis, we used MarsBar^1^ to derive for each individual subject the activity (β contrast, averaged across voxels) of the functional ROIs, defined of clusters obtained from whole-brain analyses. For ROIs identified from linear regressions in men or women alone, we tested sex differences in the correlation directly with a slope test, with age and years of education as covariates and showed two-tailed *p*-values (Zar, 1999). Note that the latter analysis did not represent “double-dipping,” as the slope tests may confirm or refute sex differences (Le et al., 2019; Dhingra et al., 2020; Ide et al., 2020). This is because the activation maps were identified with a threshold and a cluster showing correlation say in men could show a correlation that just missed the threshold in women; thus, slope tests were needed to examine whether the correlations were indeed different between the sexes.

### Mediation Analyses

We followed published routines in conducting the mediation analysis (Ide et al., 2017; Hu et al., 2018; Zhang et al., 2019; Zhornitsky et al., 2019a; Le et al., 2020; Wang et al., 2020). In a mediation analysis, the relation between the independent variable X and dependent variable Y, i.e., X→Y, is tested to see if the relation is significantly mediated by a variable M. The mediation test is performed by employing three regression equations (MacKinnon et al., 2007):

$$\begin{array}{c} Y=i_{1}+cX+e_{1}Y=i_{2}+c^{\prime }X+bM+e_{2} \end{array}$$

$$\begin{array}{c} M=i_{3}+aX+e_{3} \end{array}$$

where *a* represents X→M, *b* represents M→Y (controlling for X), *c*′ represents X→Y (controlling for M), and *c* represents X→Y. The constants i_1_, i_2_, i_3_ are the intercepts, and e_1_, e_2_, e_3_ are the residual errors. In the literature, *a*, *b*, *c* and *c*′ were referred as path coefficients or simply paths (MacKinnon et al., 2007; Wager et al., 2008), and we followed this notation. Variable M is a mediator of the correlation X→Y if (*c* –*c*′), which is mathematically equivalent to the product of the paths *a*^∗^*b*, is significantly different from zero (MacKinnon et al., 2007). If the product *a*^∗^*b* and the paths *a* and *b* are significant, one concludes that X→Y is mediated by M. In addition, if path *c*′ is not significant, there is no direct connection from X to Y and that X→Y is completely mediated by M. Note that path *b* is the relation between Y and M, controlling for X, and should not be confused with the correlation coefficient between Y and M.

As ASR subscores, regional activity, and behavioral measures (AR_*SOC*_ – AR_*RAN*_ or RT_*SOC*_ – RT_*RAN*_) may be correlated pair-wise, we performed a mediation analysis to explore their inter-relationships. Behavioral measures would be conceptually unlikely to serve as a mediating (M) or independent (X) variable, and thus we would only consider mediation models where they served as a dependent variable Y.

## Results

### Behavioral Performance in the Social Cognition Task

**FIGURE 1:**
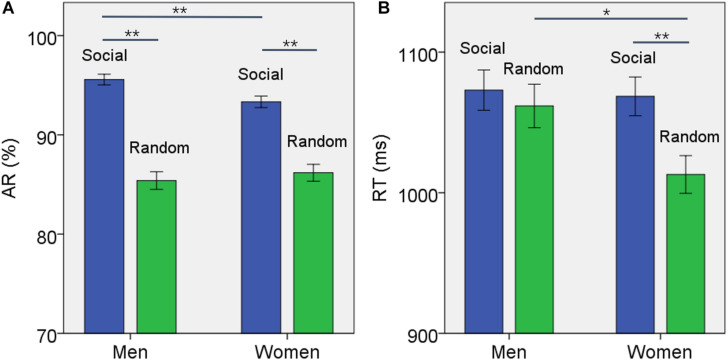
Behavioral results (mean ± SE) showed the **(A)** accuracy rate (AR) and **(B)** reaction time (RT) for social and random block each in men and women. Accuracy rate was significantly higher for social than random block in both men and women but the difference was larger for men. The social blocks showed slower RT than the random block in women but not in men. *p ≤ 0.01, **p ≤ 0.001.

For AR, the results of ANOVA showed a significant block type main effect (*F*_(1,958)_ = 155.475, *p* < 0.001), but not sex main effect (*F*_(1,958)_ = 0.891, *p* = 0.345). The AR of social block was higher than that of random block in men and women combined. In addition, the interaction of sex × block type was significant (*F*_(1,958)_ = 4.755, *p* = 0.029). Simple main effect analysis showed a significant difference both in men (*t*_457_ = 10.591, *p* < 0.001) and in women (*t*_500_ = 7.172, *p* < 0.001) (Figure 1A). Further, the difference in AR between blocks or AR_*SOC*_ – AR_*RAN*_ was significantly higher in men than in women (*t*_955_ = 2.188, *p* = 0.020).

For RT, the results of ANOVA showed a significant block type main effect (*F*_(1,958)_ = 9.394, *p* = 0.002), but not sex main effect (*F*_(1,958)_ = 2.461, *p* = 0.117). The RT of social block was longer than RT of random block in men and women combined. In addition, the interaction of sex × block type was significant (*F*_(1,958)_ = 4.134, *p* = 0.042). Simple main effect analysis showed that the RT of social block was longer than RT of random block in women (*t*_500_ = 3.736, *p* < 0.001) but not in men (*t*_457_ = 0.704, *p* = 0.482) (Figure 1B). The difference in RT between blocks or RT_*SOC*_ – RT_*RAN*_ was significantly stronger in women than in men (*t*_955_ = −2.231, *p* = 0.019).

The results of linear regression of each of the 12 ASR subscores against AR_*SOC*_ – AR_*RAN*_ and RT_*SOC*_ – RT_*RAN*_ are shown for men and women separately in Supplementary Tables S1, S2, respectively. At a *p*-value corrected for the total number of tests – 0.05/(12 subscales x 2 sexes x 2 behavioral indexes) = 0.001042, none of the regressions were significant and only 12 of 48 regressions showed an uncorrected *p* < 0.05. Further, there were no significant sex differences in the correlation for any of the syndromal subscales, either. Across all subjects, only the internalizing subscore showed a significant correlation with AR_*SOC*_ – AR_*RAN*_ (*r* = 0.115, *p* = 0.0004). The internalizing subscore did not show a significant correlation with RT_*SOC*_ – RT_*RAN*_ (*r* = 0.007, *p* = 0.839).

### Brain Activations to Social vs. Random Stimuli

In examining regional responses to social vs. random block, we first conducted a one-sample *t* test on the entire cohort and on men and women separately. Supplementary Figure S1 shows the results. Exposure to social vs. random interactions engaged a wide swath of cortical and subcortical regions, including bilateral temporal and fronto-parietal cortex, cerebellum, frontopolar cortex, hippocampus/parahippocampal gyrus, occipito-parietal sulcus, precuneus, thalamus, and caudate nucleus. In contrast, exposure to random vs. social interactions engaged higher activations in bilateral ventral medial occipital cortex, superior frontal gyri, medial and superior parietal cortex, ventromedial prefrontal cortex including the anterior cingulate gyrus, posterior cingulate cortex, right lateral orbitofrontal cortex, and anterior insula.

**FIGURE 2:**
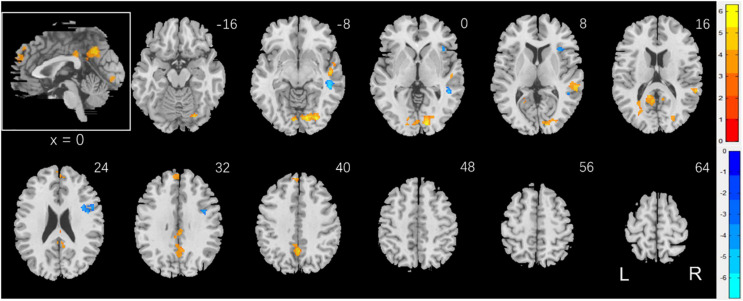
Sex differences in regional brain activations: two-sample *t*-test of the contrast (social – random) between men and women with age, years of education as covariates. Voxel *p* < 0.001, uncorrected. All clusters with cluster *p* < 0.05, corrected for family-wise error, are shown in Table 2. Color bars show voxel *t* values; warm: men > women, cool: women > men. Clusters are overlaid on a T1 structural image in neurological orientation: right = right. The inset shows a mid-sagittal section to highlight the clusters in the precuneus, posterior cingulate cortex and dorsomedial prefrontal cortex.

To examine sex differences, we conducted a two-sample *t*-test to compare men and women with age and years of education as covariates. At voxel *p* < 0.001, uncorrected, in combination with cluster-level *p* < 0.05, family-wise error corrected, men relative to women showed higher activation in the right superior temporal gyrus, bilateral occipital and posterior cingulate cortex, and precuneus (Figure 2). Women showed higher activation in the right inferior frontal cortex, as compared to men, during exposure to social vs. random interactions. These clusters are summarized in Table 2.

**TABLE 2:** Sex differences in regional activations to social vs. random stimuli.

Region	Cluster size (k)	Peak voxel (Z)	Cluster FWE *p*- value	MNI coordinate (mm)		
				X	Y	Z
*Men > Women*						
Temporal_Sup_R	180	6.26	0.000	58	−34	10
Temporal_Sup_R	150	6.25	0.000	50	−16	−4
Calcarine_R	746	5.90	0.000	14	−88	2
Precuneus_L	338	5.44	0.000	0	−56	40
L Post. Cingulate C*	252	4.59	0.000	−12	−46	18
Frontal_Sup_Medial_L	145	4.36	0.001	−8	54	30
Cingulum_Mid_R	77	4.24	0.021	2	−30	32
*Women > Men*						
R inferior frontal G*	235	5.65	0.000	42	2	26
Cerebellum_6_R	67	5.01	0.039	34	−54	−22
Insula_R	67	4.54	0.039	38	22	2

Brain regions were identified by reference to the Automated Anatomic Labeling Atlas (Tzourio-Mazoyer et al., 2002). β values are presented as mean ± SD. We referred to Duvernoy’s atlas (Duvernoy, 2009) for coordinates (*) that were not identified by the AAL. R: right; L: left; C: cortex; G: gyrus.

### Brain Activations to Social vs. Random Stimuli in Correlation With Accuracy Rate

**FIGURE 3:**
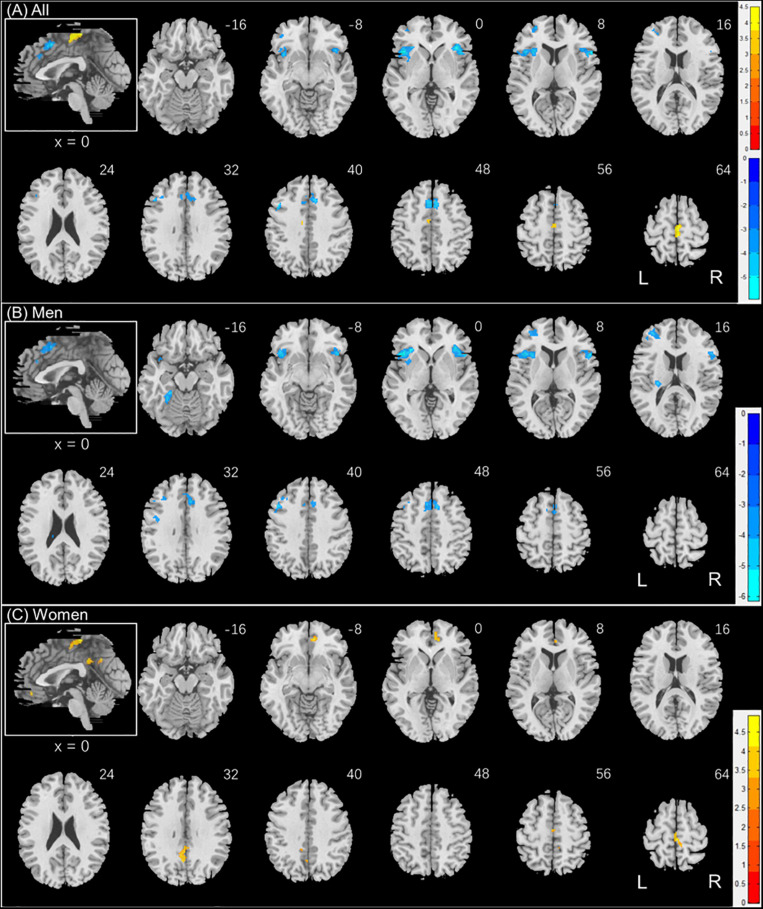
Regional responses to social interaction vs. random movement in correlation with “AR_*SOC*_ – AR_*RAN*_” in **(A)** all subjects: whole-brain regression with sex, age, and years of education as covariates; and in **(B)** men and **(C)** women separately, with age and years of education as covariates. Warm/cool color bars show clusters with positive/negative correlation with “AR_*SOC*_ – AR_*RAN*_”; *p* < 0.001, uncorrected, in combination with cluster *p* < 0.05 FWE-corrected. The insets highlight the clusters in a mid-sagittal section.

Individuals varied in how accurately they identified social vs. random interactions. We conducted a whole brain linear regression of the contrast (social vs. random) against “AR_*SOC*_ – AR_*RAN*_” across all subjects, with sex, age, and years of education as covariates. Clusters that met the threshold of voxel *p* < 0.001, uncorrected, in combination with cluster p < 0.05 FWE-corrected are shown in Figure 3A. A cluster in the paracentral lobule (PCL) showed activation in positive correlation with AR_*SOC*_ – AR_*RAN*_. Bilateral anterior insula (AI), dorsomedial prefrontal cortex in the anterior pre-supplementary motor area (preSMA), and the left middle frontal gyrus (MFG) showed higher activation to social vs. random interaction in negative correlation with AR_*SOC*_ – AR_*RAN*_.

We next conducted a whole brain linear regression of contrast (social – random) against “AR_*SOC*_ – AR_*RAN*_” for men and women separately, with age and years of education as covariates. For men alone, no clusters showed activation in positive correlation with “AR_*SOC*_ – AR_*RAN*_.” Bilateral AI, anterior preSMA, left MFG and inferior superior frontal gyrus showed activation in negative correlation with “AR_*SOC*_ – AR_*RAN*_” (Figure 3B). For women alone, a cluster each in the precuneus and in the medial orbitofrontal cortex showed activation in positive correlation with “AR_*SOC*_ – AR_*RAN*_” (Figure 3C). No clusters showed activations in negative correlation. These clusters are summarized in Table 3.

**TABLE 3:** Regional activations in correlation with AR_*SOC*_ – AR_*RAN*_.

Region	Cluster size (k)	Peak voxel (Z)	Cluster FWE *p*- value	MNI coordinate (mm)		
				X	Y	Z
*All (N = 959)*						
Paracentral Lobule*	193	+4.48	0.000	0	−18	64
Insula_R	395	−5.87	0.000	38	24	−2
Insula_L	531	−5.64	0.000	−42	18	2
PreSMA*	520	−5.13	0.000	−4	14	50
Frontal_Mid_L	162	−4.91	0.000	−44	16	40
Frontal_Mid_L	126	−4.42	0.002	−30	48	12
*Men (N = 458)*						
Insula_R	498	−6.01	0.000	38	26	−2
Insula_L	660	−5.68	0.000	−44	18	0
Frontal_Mid_L	251	−4.90	0.000	−46	18	40
Cingulum_Ant_R	472	−4.79	0.000	12	24	28
Fusiform_L	77	−4.71	0.035	−24	−38	−16
Frontal_Mid_L	287	−4.60	0.000	−30	46	12
Cerebelum_Crus1_L	86	−4.20	0.022	−34	−62	−30
*Women (N = 501)*						
Frontal_Med_Orb_R	89	+4.89	0.008	10	36	−12
Paracentral_Lobule_R	96	+4.18	0.005	2	−28	64
Precuneus_L	240	+4.13	0.000	−4	−56	34
Cingulum_Ant_R	62	+3.97	0.043	6	44	2

Brain regions were identified by reference to the Automated Anatomic Labeling Atlas (Tzourio-Mazoyer et al., 2002). We referred to Duvernoy’s atlas (Duvernoy, 2009) for coordinates (*) that were not clearly identified by the AAL. R: right; L: left; preSMA: presupplementary motor area. The sign of *Z* value shows the direction of correlation.

**FIGURE 4:**
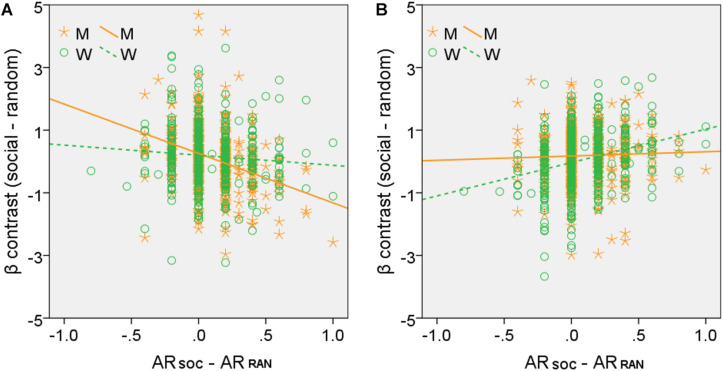
Slope tests of sex differences in the correlation between regional activities and AR_*SOC*_ – AR_*RAN*_. Each data point represents one subject. All clusters identified of **(A)** men (M, orange, clusters shown in Figure 3B) and **(B)** women (W, green, clusters shown in Figure 3C), respectively, were combined as the ROIs. Both tests showed significant sex differences: **(A)** *Z* = –3.59, *p* = 0.0003; **(B)** Z = –3.83, *p* = 0.0001.

For the brain regions showing activations in correlation with AR_*SOC*_ – AR_*RAN*_ in men or women alone, we computed the β estimate (social - random) for all of the clusters combined, as identified in men or women, respectively, for all subjects. We then tested sex differences in the correlations with slope tests (Zar, 1999), as shown in Figure 4. We also performed the same analyses for each individual cluster, and the results are shown in Supplementary Tables S3, S4, respectively.

We further examined whether the regional activities were related to ASR Internalizing scores, which, as shown earlier, were significantly correlated with AR_*SOC*_ – AR_*RAN*_ in men and women combined. Thus, for the entire sample, we performed a linear regression of the β contrast (social vs. random) against two regions of interest, each of the five “negative” clusters combined and of the single “positive” cluster in the PCL, respectively. The results showed a significant correlation of ASR Internalizing score with PCL activity (*r* = 0.090, *p* = 0.005) but not with the negative clusters (*r* = 0.029, *p* = 0.364), with age, sex, and years of education as covariates.

**FIGURE 5:**
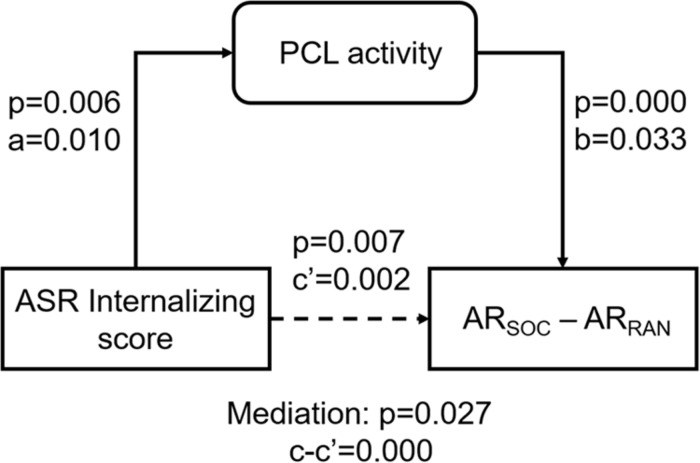
Mediation analysis. PCL activity incompletely mediated the correlation between ASR Internalizing score and AR_*SOC*_ – AR_*RAN*_. The *p*-value associated with mediation are for the path “c-c′” or “a*b.”

As ASR Internalizing score, PCL activity, and AR_*SOC*_ – AR_*RAN*_ were correlated pair-wise, we performed a mediation analysis to explore their inter-relationships. AR_*SOC*_ – AR_*RAN*_ represents a behavioral outcome measure and would be conceptually unlikely to serve as a mediating (M) or independent (X) variable. Thus, we only considered two models: (model 1; Figure 5) ASR Internalizing score → PCL activity → AR_*SOC*_ – AR_*RAN*_; and (model 2) PCL activity → ASR Internalizing score → AR_*SOC*_ – AR_*RAN*_. The results showed a significant mediation in model 1 (path c-c′ *p* = 0.027). However, the mediation was incomplete (path c′ *p* = 0.007). Model 2 did not show a significant mediation (path c-c′ *p* = 0.106).

### Brain Activations to Social vs. Random Stimuli in Correlation With Reaction Time

**FIGURE 6:**
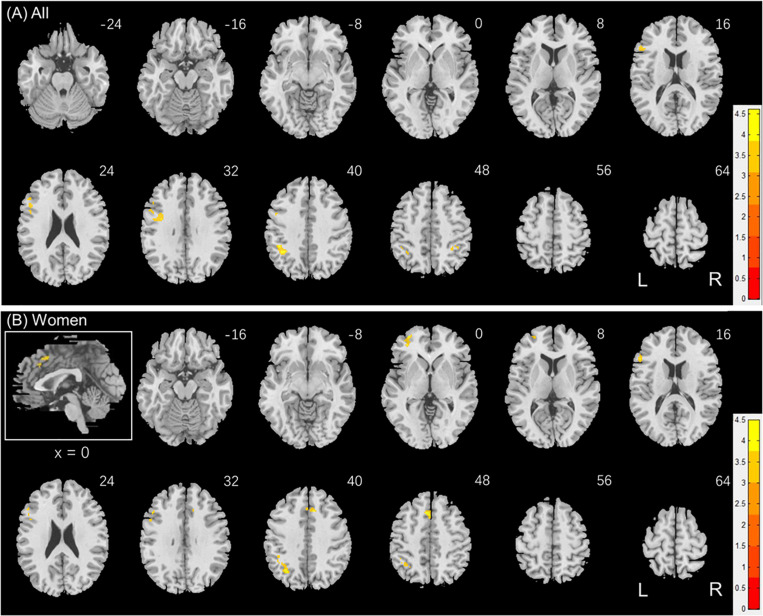
Regional responses to social interaction vs. random movement in correlation with “RT_*SOC*_ – RT_*RAN*_” in **(A)** all subjects: whole-brain regression with sex, age and years of education as covariates; and in **(B)** women, with age and years of education as covariates. Warm/cool color bars show clusters with positive/negative correlation with “RT_*SOC*_ – RT_*RAN*_”; voxel *p* < 0.001, uncorrected, in combination with cluster *p* < 0.05 FWE-corrected. The insets highlight the clusters in a mid-sagittal section.

Individuals also varied in how quickly they identified social vs. random interactions. We conducted a whole brain linear regression of the contrast (social - random) against “RT_*SOC*_ – RT_*RAN*_” across all subjects, with sex, age, and years of education as covariates. Clusters that met the threshold of voxel *p* < 0.001, uncorrected, in combination with cluster *p* < 0.05 FWE-corrected are shown in Figure 6A. Bilateral inferior parietal cortex, left inferior frontal cortex showed activation in positive correlation with RT_*SOC*_ – RT_*RAN*_. No cluster showed activation to social vs. random in negative correlation with RT_*SOC*_ – RT_*RAN*_.

We next conducted a whole brain linear regression of contrast (social – random) against “RT_*SOC*_ – RT_*RAN*_” for men and women separately, with age and years of education as covariates. For men alone, no clusters showed activation in positive or negative correlation with “RT_*SOC*_ – RT_*RAN*_.” For women alone, left supplementary motor area, left inferior parietal cortex, left middle and inferior frontal cortex showed activation in positive correlation with “RT_*SOC*_ – RT_*RAN*_” (Figure 6B).

These clusters are summarized in Table 4.

**TABLE 4:** Regional activations in correlation with RT_*SOC*_ – RT_*RAN*_.

Region	Cluster size (k)	Peak voxel (Z)	Cluster FWE *p*- value	MNI coordinate (mm)		
				X	Y	Z
*All (N = 959)*						
Parietal_Inf_L	157	4.59	0.000	−42	−42	42
Frontal_Inf_Tri_L	289	4.44	0.000	−50	26	20
Parietal_Inf_R	77	3.76	0.021	32	−48	54
*Women (N = 501)*						
Frontal_Mid_L	87	4.46	0.009	−34	50	2
Parietal_Inf_L	177	4.45	0.000	−32	−62	40
Supp_Motor_Area_L	136	4.18	0.001	−4	22	48
Frontal_Inf_Tri_L	167	4.12	0.000	−50	26	20

*Brain regions were identified by reference to the Automated Anatomic Labeling Atlas (Tzourio-Mazoyer et al., 2002). R: right; L: left; preSMA: presupplementary motor area. The sign of *Z* value shows the direction of correlation.*

Likewise, we extracted the β estimate (social vs. random) for all of the clusters combined and individually, as identified in men and women together and for women alone, for all subjects. None of β estimates showed a significant correlation with ASR internalizing score (men+women: *r* = 0.043, *p* = 0.183 with age, sex and years of education as covariates; women: *r* = 0.025, *p* = 0.431, with age and years of education as covariates).

## Discussion

We showed that exposure to interacting vs. random stimuli engaged a wide array of cortical and subcortical structures, with activity of the PCL in positive correlation, and activity of bilateral AI and anterior preSMA, and left MFG in negative correlation, with the accuracy in identifying interacting vs. random stimuli. We showed that men relative to women more accurately perceived socially vs. randomly interacting stimuli. Men and women also demonstrated significant differences in regional brain activations during exposure to social vs. random stimuli and in link with individual differences in the accuracy in identifying these stimuli. Largely reflecting the findings in men and women combined, men alone showed activation in negative correlation with “AR_*SOC*_ – AR_*RAN*_” in bilateral AI, anterior preSMA, and left MFG. In contrast, women alone showed activation in the PCL, precuneus and medial orbitofrontal cortex in positive correlation with “AR_*SOC*_ – AR_*RAN*_.” The sex differences in these correlations were confirmed by slope tests. Further, we showed that, in men and women combined, PCL activity mediated the relationship between internalizing traits and AR_*SOC*_ – AR_*RAN*_, suggesting that individuals who were socially avoidant and anxiety-prone were more accurate in identifying social vs. random stimuli and this correlation was supported by the somatosensory cortex. We highlight the main findings in the discussions below.

### Sex Differences in Regional Responses to Social vs. Random Interactions

It is not entirely clear why men demonstrated higher accuracy in recognizing social interaction than women. It is important to point out that men and women did not differ in the accuracy in recognizing randomly interacting stimuli and the sex difference results from men’s higher accuracy in recognizing simulated social interactions. One possibility may have to do with men’s superior ability in perceiving motion stimuli (Murray et al., 2018). The latter study showed that, in comparison to men, women regularly took longer in reporting the direction of movement of black and white bars on a screen. This difference could not be attributed to differences in the speed of visual processing, overall visual discrimination abilities, or potential motor-related factors. In fact, the differences were not reflected in regional brain responses to visual motion stimuli, suggesting that non-visual areas may be involved in manifesting the sex differences. On the other hand, in the current study, men relative to women demonstrated a significantly smaller RT difference in recognizing social than random interaction, largely because of prolonged RT during the random interaction blocks. Together, these findings suggest that men were perhaps trying to “make sense of” the randomly interacting stimuli, thus taking longer to respond, but were better able to distinguish social from random interactions.

We showed sex differences in regional activations in recognizing social vs. random interactions. The right superior temporal gyrus (STG) responded more strongly in men than in women during social vs. random blocks, suggesting its roles in passive observation of social interactions and socially salient stimuli in men. These roles of the STG have been shown in prior studies although sex differences have not been examined (Singer et al., 2004; Schilbach et al., 2006). In contrast, a previous study showed stronger activation in women in the STG during processing of self-related social interactions (Schulte-Ruther et al., 2008). Thus, the STG may play dissociable roles in the perception of other- and self- related social interactions between the sexes. Men in comparison to women also showed higher activation in the posterior cingulate cortex, a key region of the default mode network involved in social cognition and associated with a more outward/other- vs. inward/self- directed focus during interpersonal processing (Johnson et al., 2006; Jung et al., 2015).

On the other hand, women compared to men showed higher activation in the right inferior frontal cortex and insula, which partake in evaluation of intentions in interpersonal interactions (Liu et al., 2016) and other complex social functions such as empathy (Singer et al., 2009; Gu et al., 2012), respectively. These findings indicate that women, as compared to men, may be more engaged in interpreting social interactions (Proverbio et al., 2010), an attribute related to their superiority in empathy (Baron-Cohen and Wheelwright, 2004). It has been posited that the mirror-neuron system, involving the premotor and inferior parietal cortex, provides the basic mechanism for social cognition (Rizzolatti and Craighero, 2004; Cross et al., 2009). A previous study showed that women but not men displayed stronger activity in the mirror-neuron system when watching hand actions vs. observing moving dots (Cheng et al., 2008). Therefore, we speculate that women showed lower accuracy in identifying social vs. random interactions in the current behavioral task only because the simulated interactions failed to fully and reliably engage the mirror-neuron system.

### Performance-Specific Regional Activations and Sex Differences

Higher AR_*SOC*_ – AR_*RAN*_ indicates greater sensitivity to simulated social interactions. We showed regional activations in either positive or negative correlation with AR_*SOC*_ – AR_*RAN*_ as well as sex differences in these regional responses. These data shed light on the neural mechanisms underlying the perception of social interactions. In men and women together, the activation in the PCL was positively correlated with AR_*SOC*_ – AR_*RAN*,_ and the PCL has been shown to be engaged in predicting and monitoring the outcomes of decisions during social interactions (Apps et al., 2013). Our findings thus add to this earlier study and suggest a direct relationship between PCL activity and individual capacity in perceiving social interactions. Interestingly, previous work of diffusion tensor imaging showed that, whereas the white matter integrity of the PCL increased with age in neurotypical children, those with autism spectrum disorders (ASD) showed the opposite, during adolescence (Cheng et al., 2010). Compared to healthy controls, individuals with high-functioning autism showed diminished thickness of the PCL (Scheel et al., 2011). A more recent study has implicated dynamic connectivity dysfunction of the PCL in the ASD (Fu et al., 2019). Along with these earlier findings, the current results suggest an important role of the PCL in individual capacity in perceiving social interactions and PCL dysfunction as a potential mechanism of social interaction deficits in ASD.

In contrast, regional activities negatively correlated with AR_*SOC*_ – AR_*RAN*_ involved bilateral AI, preSMA and left MFG. These regions have been associated with emotional awareness and interpersonal emotion regulation (Grecucci et al., 2013). Unlike the PCL where performance-related activities did not appear to differ between men and women (Supplementary Table S4, cluster x = 2, y = −28, z = 64), AI, preSMA and MFG showed activities in negative correlation with AR_*SOC*_ – AR_*RAN*_, primarily in men. Along with earlier discussion that men relative to women perhaps were more engaged in outward/other- directed processes when exposed to these stimuli, we speculate that disengagement from these emotion-regulatory processes may conduce to the accuracy in identifying socially vs. randomly interacting stimuli.

### The Influences of Internalizing Traits on Behavioral Performance and Regional Responses

Participants in the HCP were evaluated with many clinical instruments and we focused on the Achenbach Self Report Scale, which covered a wide array of syndromal traits. Both men and women who showed higher internalizing traits were more accurate in identifying socially vs. randomly interacting stimuli, suggesting heightened sensitivity to social interactions. The internalizing subscore, which captured a realm of psychopathology, including anxiety and social withdrawal, was also significantly correlated with regional responses to AR_*SOC*_ – AR_*RAN*_. These pair-wise correlations allowed us to perform a mediation analysis, which showed that activity of the PCL, in the somatomotor cortex, mediated the correlation between internalizing trait and AR_*SOC*_ – AR_*RAN*_. Thus, individuals who were more socially withdrawn and anxious engaged to a greater extent the PCL in monitoring social interaction and more accurately identified social vs. random interacting stimuli. Note that, as PCL activity did not demonstrate sex differences in the correlation with performance, we did not test whether its mediating role may differ between men and women. More studies are clearly needed to investigate how internalizing traits may partake in social perception and cognition differently between the sexes.

## Conclusion

A number of limitations need to be considered for the study. First, the use of geometric objects simulating social interaction had the advantage of controlling for the sex differences in processing facial emotions. On the other hand, despite the “causal nature” of the interactions, the simulated movements of geometric objects may not capture the very essence of social interactions without actually involving humans. A potentially useful alternative is to employ video clips of lights placed on actors’ limbs and joints so human motion and interaction can be clearly discerned yet without involving facial emotions. Secondly, the current findings pertain largely to passive observation of social interactions (i.e., other-related processing). It remains to be seen how men and women may differ in perceiving and evaluating social interactions that involve self-related processing and in the neural processes that underlie these more ubiquitous and important social interaction processes. Finally, multiple tests were conducted, and we corrected for the number of tests in evaluating the statistical significance of the findings within each set but not across sets of analyses.

To conclude, we demonstrated how men and women may differ in the perception of simulated social interactions and the cerebral responses that underlie these differences. Men were superior in accurately identifying social vs. random interactions, likely as a result of their predominantly other-directed psychological processes and “passivity” in observing the interactions. Nonetheless, for both men and women, those who engaged higher activity of the PCL were more accurate in identifying social interactions. The latter finding add to the literature implicating the PCL as a neural marker of dysfunctional social interaction in autism.

## Data Availability Statement

Publicly available datasets were analyzed in this study. This data can be found here: “https://wiki.humanconnectome.org/display/PublicData/HCP+Data+Dictionary+Public-+Updated+for+ the+1200+Subject+Release#HCPDataDictionaryPublic-Upda tedforthe1200SubjectRelease-Instrument:SocialRelationships.”

## Ethics Statement

The studies involving human participants were reviewed and approved by Washington University Institutional Review Board. The patients/participants provided their written informed consent to participate in this study.

## Author Contributions

GL, XT, and C-SL contributed to the conceptualization of the study. GL performed data analyses. YC, WW, ID, SZ, and XT contributed to critical evaluation of the data. All authors contributed to the writing and revisions of the manuscript.

## Conflict of Interest

The authors declare that the research was conducted in the absence of any commercial or financial relationships that could be construed as a potential conflict of interest.

## Footnotes

1 http://marsbar.sourceforge.net/

## References

[B1] Achenbach T. M. (2009). *The Achenbach System of Empirically Based Assessemnt (ASEBA): Development, Findings, Theory, and Applications.* Burlington, VT: University of Vermont Research Center for Children, Youth, & Families.

[B2] Akitsuki Y. Decety J. (2009). Social context and perceived agency affects empathy for pain: an event-related fMRI investigation. *Neuroimage* 47 722–734. 10.1016/j.neuroimage.2009.04.091 19439183

[B3] APA (2013). *Diagnostic and Statistical Manual of Mental Disorders*, 5th Edn Arlington, TX: American Psychiatric Association.

[B4] Apps M. A. Lockwood P. L. Balsters J. H. (2013). The role of the midcingulate cortex in monitoring others’ decisions. *Front. Neurosci.* 7:251. 10.3389/fnins.2013.00251 24391534 PMC3868891

[B5] Arioli M. Perani D. Cappa S. Proverbio A. M. Zani A. Falini A. (2018). Affective and cooperative social interactions modulate effective connectivity within and between the mirror and mentalizing systems. *Hum. Brain Mapp.* 39 1412–1427. 10.1002/hbm.23930 29265483 PMC6866589

[B6] Babchuk W. A. Hames R. B. Thompson R. A. (1985). Sex differences in the recognition of infant facial expressions of emotion: the primary caretaker hypothesis. *Ethol. Sociobiol.* 6 89–101. 10.1016/0162-3095(85)90002-0

[B7] Barch D. M. Burgess G. C. Harms M. P. Petersen S. E. Schlaggar B. L. Corbetta M. (2013). Function in the human connectome: task-fMRI and individual differences in behavior. *Neuroimage* 80 169–189. 10.1016/j.neuroimage.2013.05.033 23684877 PMC4011498

[B8] Baron-Cohen S. Wheelwright S. (2004). The empathy quotient: an investigation of adults with asperger syndrome or high functioning autism, and normal sex differences. *J. Autism. Dev. Disord.* 34 163–175. 10.1023/b:jadd.0000022607.19833.00 15162935

[B9] Bornstein M. H. Hahn C. S. Haynes O. M. (2010). Social competence, externalizing, and internalizing behavioral adjustment from early childhood through early adolescence: developmental cascades. *Dev. Psychopathol.* 22 717–735. 10.1017/s0954579410000416 20883577 PMC3412561

[B10] Buss D. M. (1995). Psychological sex differences: origins through sexual selection. *Am. Psychol.* 50 164–168. 10.1037/0003-066x.50.3.164 7726470

[B11] Castelli F. Happe F. Frith U. Frith C. (2000). Movement and mind: a functional imaging study of perception and interpretation of complex intentional movement patterns. *Neuroimage* 12 314–325. 10.1006/nimg.2000.0612 10944414

[B12] Centelles L. Assaiante C. Nazarian B. Anton J. L. Schmitz C. (2011). Recruitment of both the mirror and the mentalizing networks when observing social interactions depicted by point-lights: a neuroimaging study. *PLoS One* 6:e15749. 10.1371/journal.pone.0015749 21249224 PMC3018423

[B13] Cheng Y. Chou K. H. Chen I. Y. Fan Y. T. Decety J. Lin C. P. (2010). Atypical development of white matter microstructure in adolescents with autism spectrum disorders. *Neuroimage* 50 873–882. 10.1016/j.neuroimage.2010.01.011 20074650

[B14] Cheng Y. Lee P. L. Yang C. Y. Lin C. P. Hung D. Decety J. (2008). Gender differences in the mu rhythm of the human mirror-neuron system. *PLoS One* 3:e2113. 10.1371/journal.pone.0002113 18461176 PMC2361218

[B15] Cross E. S. Kraemer D. J. Hamilton A. F. Kelley W. M. Grafton S. T. (2009). Sensitivity of the action observation network to physical and observational learning. *Cereb. Cortex* 19 315–326. 10.1093/cercor/bhn083 18515297 PMC2638791

[B16] Deuse L. Rademacher L. M. Winkler L. Schultz R. T. Grunder G. Lammertz S. E. (2016). Neural correlates of naturalistic social cognition: brain-behavior relationships in healthy adults. *Soc. Cogn. Affect. Neurosci.* 11 1741–1751. 10.1093/scan/nsw094 27496338 PMC5091685

[B17] Dhingra I. Zhang S. Zhornitsky S. Le T. M. Wang W. Chao H. H. (2020). The effects of age on reward magnitude processing in the monetary incentive delay task. *Neuroimage* 207 116368. 10.1016/j.neuroimage.2019.116368 31743790 PMC7463276

[B18] Duvernoy H. M. (2009). *The Human Brain*,edition2ed Edn. New York, NY: Springer-Verlag.

[B19] Eggum N. D. Eisenberg N. Spinrad T. L. Valiente C. Edwards A. Kupfer A. S. (2009). Predictors of withdrawal: possible precursors of avoidant personality disorder. *Dev. Psychopathol.* 21 815–838. 10.1017/s0954579409000443 19583885 PMC2774890

[B20] Fischer A. H. Kret M. E. Broekens J. (2018). Gender differences in emotion perception and self-reported emotional intelligence: a test of the emotion sensitivity hypothesis. *PLoS One* 13:e0190712. 10.1371/journal.pone.0190712 29370198 PMC5784910

[B21] Fu Z. Tu Y. Di X. Du Y. Sui J. Biswal B. B. (2019). Transient increased thalamic-sensory connectivity and decreased whole-brain dynamism in autism. *Neuroimage* 190 191–204. 10.1016/j.neuroimage.2018.06.003 29883735 PMC6281849

[B22] Grecucci A. Giorgetta C. Bonini N. Sanfey A. G. (2013). Reappraising social emotions: the role of inferior frontal gyrus, temporo-parietal junction and insula in interpersonal emotion regulation. *Front. Hum. Neurosci.* 7:523. 10.3389/fnhum.2013.00523 PMC3759791 24027512

[B23] Gu X. Gao Z. Wang X. Liu X. Knight R. T. Hof P. R. (2012). Anterior insular cortex is necessary for empathetic pain perception. *Brain* 135 2726–2735. 10.1093/brain/aws199 22961548 PMC3437027

[B24] Hall J. A. (1978). Gender effects in decoding nonverbal cues. *Psychol. Bull.* 85 845–857. 10.1037/0033-2909.85.4.845

[B25] Hu S. Ide J. S. Chao H. H. Castagna B. Fischer K. A. Zhang S. (2018). Structural and functional cerebral bases of diminished inhibitory control during healthy aging. *Hum. Brain Mapp.* 39 5085–5096. 10.1002/hbm.24347 30113124 PMC6287913

[B26] Ide J. S. Li H. T. Chen Y. Le T. M. Li C. S. P. Zhornitsky S. (2020). Gray matter volumetric correlates of behavioral activation and inhibition system traits in children: an exploratory voxel-based morphometry study of the ABCD project data. *Neuroimage* 220:117085. 10.1016/j.neuroimage.2020.117085 32592852 PMC7572877

[B27] Ide J. S. Zhornitsky S. Hu S. Zhang S. Krystal J. H. Li C. S. R. (2017). Sex differences in the interacting roles of impulsivity and positive alcohol expectancy in problem drinking: a structural brain imaging study. *NeuroImage* 14 750–759. 10.1016/j.nicl.2017.03.015 28413777 PMC5385596

[B28] Johnson M. K. Raye C. L. Mitchell K. J. Touryan S. R. Greene E. J. Nolen-Hoeksema S. (2006). Dissociating medial frontal and posterior cingulate activity during self-reflection. *Soc. Cogn. Affect. Neurosci.* 1 56–64. 10.1093/scan/nsl004 18574518 PMC2435374

[B29] Jung M. Mody M. Saito D. N. Tomoda A. Okazawa H. Wada Y. (2015). Sex differences in the default mode network with regard to autism spectrum traits: a resting state fMRI study. *PLoS One* 10:e0143126. 10.1371/journal.pone.0143126 26600385 PMC4658035

[B30] Klonsky E. D. Jane J. S. Turkheimer E. Oltmanns T. F. (2002). Gender role and personality disorders. *J. Personal. Disord.* 16 464–476. 10.1521/pedi.16.5.464.22121 12489312 PMC4364134

[B31] Kret M. E. De Gelder B. (2012). A review on sex differences in processing emotional signals. *Neuropsychologia* 50 1211–1221. 10.1016/j.neuropsychologia.2011.12.022 22245006

[B32] Kujala M. V. Carlson S. Hari R. (2012). Engagement of amygdala in third-person view of face-to-face interaction. *Hum. Brain Mapp.* 33 1753–1762. 10.1002/hbm.21317 21674692 PMC6870477

[B33] Le T. M. Chao H. Levy I. Li C. S. R. (2020). Age-related changes in the neural processes of reward-directed action and inhibition of action. *Front. Psychol.* 11:1121. 10.3389/fpsyg.2020.01121 32587547 PMC7298110

[B34] Le T. M. Wang W. Zhornitsky S. Dhingra I. Zhang S. Li C. S. R. (2019). Reward sensitivity and electrodermal responses to actions and outcomes in a go/no-go task. *PLoS One* 14:e0219147. 10.1371/journal.pone.0219147 31344045 PMC6657849

[B35] Lee K. H. Boltz M. Lee H. Algase D. L. (2017). Does social interaction matter psychological well-being in persons with dementia? *Am. J. Alzheimers Dis. Other Demen.* 32 207–212. 10.1177/1533317517704301 28417644 PMC10852833

[B36] Li G. Zhang S. Le T. M. Tang X. Li C. S. R. (2020). Neural responses to reward in a gambling task: sex differences and individual variation in reward-driven impulsivity. *Cereb. Cortex Commun.* 1:tgaa025. 10.1093/texcom/tgaa025 PMC7446303 32864617

[B37] Liu T. Saito H. Oi M. (2016). Obstruction increases activation in the right inferior frontal gyrus. *Soc. Neurosci.* 11 344–352. 10.1080/17470919.2015.1088469 26366676

[B38] MacKinnon D. P. Fairchild A. J. Fritz M. S. (2007). Mediation analysis. *Annu. Rev. Psychol.* 58 593–614. 16968208 10.1146/annurev.psych.58.110405.085542 PMC2819368

[B39] Maestripieri D. Pelka S. (2002). Sex differences in interest in infants across the lifespan. *Hum. Nat.* 13:327. 10.1007/s12110-002-1018-1 26192926

[B40] Murray S. O. Schallmo M. P. Kolodny T. Millin R. Kale A. Thomas P. (2018). Sex differences in visual motion processing. *Curr. Biol.* 28 2794.e3–2799.e3. 30122530 10.1016/j.cub.2018.06.014 PMC6133755

[B41] Pavlova M. (2017). ex and gender affect the social brain: beyond simplicity. *J. Neurosci. Res.* 95 235–250. 10.1002/jnr.23871 27688155

[B42] Pavlova M. Guerreschi M. Lutzenberger W. Sokolov A. N. Krageloh-Mann I. (2010). Cortical response to social interaction is affected by gender. *Neuroimage* 50 1327–1332. 10.1016/j.neuroimage.2009.12.096 20056153

[B43] Poldrack R. A. Fletcher P. C. Henson R. N. Worsley K. J. Brett M. Nichols T. E. (2008). Guidelines for reporting an fMRI study. *Neuroimage* 40 409–414. 10.1016/j.neuroimage.2007.11.048 18191585 PMC2287206

[B44] Powers K. E. Chavez R. S. Heatherton T. F. (2016). Individual differences in response of dorsomedial prefrontal cortex predict daily social behavior. *Soc. Cogn. Affect. Neurosci.* 11 121–126. 10.1093/scan/nsv096 26206505 PMC4692321

[B45] Proverbio A. M. Riva F. Zani A. (2010). When neurons do not mirror the agent’s intentions: sex differences in neural coding of goal-directed actions. *Neuropsychologia* 48 1454–1463. 10.1016/j.neuropsychologia.2010.01.015 20117123

[B46] Rasmussen C. E. Jiang Y. V. (2019). Judging social interaction in the Heider and Simmel movie. *Q. J. Exp. Psychol.* 72 2350–2361. 10.1177/1747021819838764 30827187

[B47] Rizzolatti G. Craighero L. (2004). The mirror-neuron system. *Annu. Rev. Neurosci.* 27 169–192. 15217330 10.1146/annurev.neuro.27.070203.144230

[B48] Sapey-Triomphe L. A. Centelles L. Roth M. Fonlupt P. Henaff M. A. Schmitz C. (2017). Deciphering human motion to discriminate social interactions: a developmental neuroimaging study. *Soc. Cogn. Affect. Neurosci.* 12 340–351. 10.1093/scan/nsw117 28008075 PMC5390742

[B49] Scheel C. Rotarska-Jagiela A. Schilbach L. Lehnhardt F. G. Krug B. Vogeley K. (2011). Imaging derived cortical thickness reduction in high-functioning autism: key regions and temporal slope. *Neuroimage* 58 391–400. 10.1016/j.neuroimage.2011.06.040 21749926

[B50] Schilbach L. Wohlschlaeger A. M. Kraemer N. C. Newen A. Shah N. J. Fink G. R. (2006). Being with virtual others: neural correlates of social interaction. *Neuropsychologia* 44 718–730. 10.1016/j.neuropsychologia.2005.07.017 16171833

[B51] Schulte-Ruther M. Markowitsch H. J. Shah N. J. Fink G. R. Piefke M. (2008). Gender differences in brain networks supporting empathy. *Neuroimage* 42 393–403. 10.1016/j.neuroimage.2008.04.180 18514546

[B52] Simpson E. A. Nicolini Y. Shetler M. Suomi S. J. Ferrari P. F. Paukner A. (2016). Experience-independent sex differences in newborn macaques: females are more social than males. *Sci. Rep.* 6:19669. 10.1038/srep19669 PMC4726418 26794858

[B53] Singer T. Critchley H. D. Preuschoff K. (2009). A common role of insula in feelings, empathy and uncertainty. *Trends Cogn. Sci.* 13 334–340. 10.1016/j.tics.2009.05.001 19643659

[B54] Singer T. Kiebel S. J. Winston J. S. Dolan R. J. Frith C. D. Stefan J. K. (2004). Brain responses to the acquired moral status of faces. *Neuron* 41 653–662. 10.1016/s0896-6273(04)00014-5 14980212

[B55] Tzourio-Mazoyer N. Landeau B. Papathanassiou D. Crivello F. Etard O. Delcroix N. (2002). Automated anatomical labeling of activations in SPM using a macroscopic anatomical parcellation of the MNI MRI single-subject brain. *Neuroimage* 15 273–289. 10.1006/nimg.2001.0978 11771995

[B56] Wager T. D. Davidson M. L. Hughes B. L. Lindquist M. A. Ochsner K. N. (2008). Prefrontal-subcortical pathways mediating successful emotion regulation. *Neuron* 59 1037–1050. 10.1016/j.neuron.2008.09.006 18817740 PMC2742320

[B57] Walbrin J. Downing P. Koldewyn K. (2018). Neural responses to visually observed social interactions. *Neuropsychologia* 112 31–39. 10.1016/j.neuropsychologia.2018.02.023 29476765 PMC5899757

[B58] Wang W. Zhornitsky S. Le T. M. Zhang S. Li C. R. (2019). Heart rate variability, cue-evoked ventromedial prefrontal cortical response, and problem alcohol use in adult drinkers. *Biol. Psychiatry Cogn. Neurosci. Neuroimaging* 5 619–628. 10.1016/j.bpsc.2019.12.013 32061544 PMC7286768

[B59] Wang W. Zhornitsky S. Le T. M. Zhang S. Li C.-S. R. (2020). Heart rate variability, cue-evoked ventromedial prefrontal cortical response, and problem alcohol use in adult drinkers. *Bio. Psychiatry* 5 619–628. 10.1016/j.bpsc.2019.12.013 PMC7286768 32061544

[B60] Wheatley T. Milleville S. C. Martin A. (2007). Understanding animate agents: distinct roles for the social network and mirror system. *Psychol.Sci.* 18 469–474. 10.1111/j.1467-9280.2007.01923.x 17576256

[B61] Wood W. Eagly A. H. (2002). A cross-cultural analysis of the behavior of women and men: implications for the origins of sex differences. *Psychol. Bull.* 128 699–727. 10.1037/0033-2909.128.5.699 12206191

[B62] Zar J. H. (1999). *Biostatistical Analysis.* Upper Saddle Rive, NJ: Prentice Hall.

[B63] Zhang S. Zhornitsky S. Le T. M. Li C.-S. R. (2019). Hypothalamic responses to cocaine and food cues in individuals with cocaine dependence. *Int. J. Neuropsychopharmacol.* 22 754–764. 10.1093/ijnp/pyz044 31420667 PMC6929672

[B64] Zhornitsky S. Zhang S. Ide J. S. Chao H. H. Wang W. Le T. M. (2019a). Alcohol expectancy and cerebral responses to cue-elicited craving in adult nondependent drinkers. *Biol. Psychiatry Cogn. Neurosci. Neuroimaging* 4 493–504. 10.1016/j.bpsc.2018.11.012 30711509 PMC6500759

[B65] Zhornitsky S. Zhang S. Ide J. S. Chao H. H. Wang W. Le T. M. (2019b). Alcohol expectancy and cerebral responses to cue-elicited craving in adult nondependent drinkers. *Biol. Psychiatry Cogn. Neurosci. Neuroimaging* 4 493–504. 30711509 10.1016/j.bpsc.2018.11.012 PMC6500759

