# Supplementary material for: Sex Differences in Neural Responses to the Perception of Social Interactions
Source: Front Hum Neurosci. 2020 Sep 11;14:565132. doi: 10.3389/fnhum.2020.565132 (PMC7518190; doi:10.3389/fnhum.2020.565132)
Supplement: Supplementary file 1 [file Data_Sheet_1.docx]

**Supplement:**

**Li et al.,** **Sex differences in neural responses to the perception of social interactions**

**Supplementary Table S1.** Correlation between AR_SOC_ – AR_RAN_ and Achenbach Adult Self-Report Syndrome Scale raw score

| Characteristic | | All (n=959) | | | Men (n = 458) | | Women (n = 501) | | Slope test | | | | |
| --- | --- | --- | --- | --- | --- | --- | --- | --- | --- | --- | --- | --- | --- |
|  |  | r | | *p* | r | *p* | r | *p* | *Z* | | | | *p* |
| ASR_Anxd | 0.100 | | 0.002 | | 0.072 | 0.124 | 0.120 | 0.007 | -0.75 | | | | 0.4533 |
| ASR_Witd | 0.110 | | 0.001 | | 0.098 | 0.036 | 0.120 | 0.007 | -0.34 | | | 0.7339 | |
| ASR_Soma | 0.088 | | 0.007 | | 0.088 | 0.060 | 0.084 | 0.061 | 0.06 | | 0.9522 | | |
| ASR_Thot | 0.110 | | 0.001 | | 0.141 | 0.002 | 0.077 | 0.087 | 1.00 | 0.3173 | | | |
| ASR_Attn | 0.106 | | 0.001 | | 0.116 | 0.013 | 0.095 | 0.034 | 0.33 | 0.7414 | | | |
| ASR_Aggr | 0.064 | | 0.048 | | 0.071 | 0.128 | 0.055 | 0.222 | 0.25 | 0.8026 | | | |
| ASR_Rule | 0.087 | | 0.007 | | 0.063 | 0.176 | 0.114 | 0.011 | -0.79 | 0.4295 | | | |
| ASR_Intr | 0.066 | | 0.042 | | 0.105 | 0.024 | 0.030 | 0.509 | 1.16 | 0.2460 | | | |
| ASR_Oth | 0.060 | | 0.062 | | 0.075 | 0.112 | 0.042 | 0.349 | 0.51 | 0.6101 | | | |
| ASR_Crit | 0.100 | | 0.002 | | 0.122 | 0.009 | 0.075 | 0.096 | 0.73 | 0.4654 | | | |
| ASR_Intn | 0.115 | | 0.000 | | 0.095 | 0.042 | 0.128 | 0.004 | -0.52 | 0.6031 | | | |
| ASR_Extn | 0.091 | | 0.005 | | 0.098 | 0.037 | 0.083 | 0.063 | 0.23 | 0.8181 | | | |

Note: Anxious/Depressed score (ASR_Anxd), Withdrawn score (ASR_Witd), Somatic Complaints score (ASR_Soma), Thought Problems score (ASR_Thot), Attention Problems score (ASR_Attn), Aggressive Behavior score (ASR_Aggr), Rule Breaking Behavior score (ASR_Rule), Intrusive score (ASR_Intr), Other Problems score (ASR_Oth), Critical Items score (ASR_Crit), Internalizing score (ASR_Intn), Externalizing score (ASR_Extn). r and p values of linear regressions with age, sex, and years of education for all, and with age and years of education for men and women alone.

**Supplementary Table S2.** Correlation between RT_SOC_ – RT_RAN_ and Achenbach Adult Self-Report Syndrome Scale raw score

| Characteristic | | All (n=959) | | | Men (n = 458) | | Women (n = 501) | | Slope test | | | | |
| --- | --- | --- | --- | --- | --- | --- | --- | --- | --- | --- | --- | --- | --- |
|  |  | r | | *p* | r | *p* | r | *p* | *Z* | | | | *p* |
| ASR_Anxd | 0.023 | | 0.470 | | 0.037 | 0.435 | 0.019 | 0.674 | 0.28 | | | | 0.7795 |
| ASR_Witd | -0.006 | | 0.860 | | -0.021 | 0.660 | 0.018 | 0.690 | -0.6 | | | 0.5485 | |
| ASR_Soma | -0.018 | | 0.582 | | -0.067 | 0.154 | 0.029 | 0.523 | -1.48 | | 0.1389 | | |
| ASR_Thot | -0.015 | | 0.652 | | -0.058 | 0.217 | 0.040 | 0.369 | -1.51 | 0.1310 | | | |
| ASR_Attn | 0.000 | | 0.997 | | 0.017 | 0.718 | -0.011 | 0.799 | 0.43 | 0.6672 | | | |
| ASR_Aggr | 0.004 | | 0.911 | | 0.032 | 0.491 | -0.019 | 0.671 | 0.79 | 0.4295 | | | |
| ASR_Rule | -0.002 | | 0.957 | | 0.018 | 0.703 | -0.019 | 0.677 | 0.57 | 0.5687 | | | |
| ASR_Intr | -0.030 | | 0.357 | | -0.040 | 0.389 | -0.020 | 0.652 | -0.31 | 0.7566 | | | |
| ASR_Oth | 0.011 | | 0.734 | | 0.013 | 0.780 | 0.020 | 0.658 | -0.11 | 0.9124 | | | |
| ASR_Crit | 0.021 | | 0.522 | | -0.024 | 0.605 | 0.080 | 0.075 | -1.61 | 0.1074 | | | |
| ASR_Intn | 0.007 | | 0.839 | | -0.004 | 0.935 | 0.025 | 0.573 | -0.45 | 0.6527 | | | |
| ASR_Extn | -0.009 | | 0.787 | | 0.011 | 0.818 | -0.024 | 0.587 | 0.54 | 0.5892 | | | |

Note: Anxious/Depressed score (ASR_Anxd), Withdrawn score (ASR_Witd), Somatic Complaints score (ASR_Soma), Thought Problems score (ASR_Thot), Attention Problems score (ASR_Attn), Aggressive Behavior score (ASR_Aggr), Rule Breaking Behavior score (ASR_Rule), Intrusive score (ASR_Intr), Other Problems score (ASR_Oth), Critical Items score (ASR_Crit), Internalizing score (ASR_Intn), Externalizing score (ASR_Extn). r and p values of linear regressions with age, sex, and years of education for all, and with age and years of education for men and women alone.

**Supplementary Table S3.** Sex differences in the β estimate (social – random) of the ROIs identified from regression with AR_SOC_ – AR_RAN_ (Figure 3A, B, and C).

| All/individual clusters | β  Men (n=458) | | β  Women (n=501) | | T test* | |
| --- | --- | --- | --- | --- | --- | --- |
|  |  |  |  |  | T_957_ | p |
| *All - positive* |  |  |  |  |  |  |
| 0 -18 64 | -0.117 ± 1.040 | | -0.125 ± 0.952 | | 0.132 | 0.895 |
| *All - negative* |  |  |  |  |  |  |
| All clusters | -0.009 ± 1.103 | | 0.106 ± 0.901 | | -1.753 | 0.080 |
| -30 48 12 | -0.219 ± 1.622 | | -0.254 ± 1.383 | | 0.358 | 0.720 |
| -42 18 2 | 0.204 ± 1.485 | | 0.277 ± 1.007 | | -0.896 | 0.371 |
| -44 16 40 | 0.184 ± 1.699 | | 0.207 ± 1.296 | | -0.236 | 0.814 |
| -4 14 50 | -0.308 ± 1.104 | | -0.124 ± 1.035 | | -2.664 | 0.008 |
| 38 24 -2 | 0.086 ± 1.105 | | 0.250 ± 0.972 | | -2.446 | 0.015 |
| *Men* |  |  |  |  |  |  |
| All clusters | 0.095 ± 1.058 | | 0.177 ± 0.854 | | -1.313 | 0.190 |
| -24 -38 -16 | 0.801 ± 0.876 | | 0.790 ± 0.862 | | 0.194 | 0.847 |
| -30 46 12 | -0.355 ± 1.778 | | -0.314 ± 1.439 | | -0.393 | 0.695 |
| -34 -62 -30 | 0.413 ± 1.001 | | 0.411 ± 0.921 | | 0.030 | 0.976 |
| -44 18 0 | 0.262 ± 1.461 | | 0.311 ± 1.008 | | -0.607 | 0.544 |
| -46 18 40 | 0.265 ± 1.398 | | 0.245 ± 1.159 | | -0.235 | 0.814 |
| 12 24 28 | -0.242 ± 1.107 | | -0.057 ± 1.027 | | -2.695 | 0.007 |
| 38 26 -2 | 0.207 ± 1.148 | | 0.357 ± 1.017 | | -2.136 | 0.033 |
| *Women* |  |  |  |  |  |  |
| All clusters | 0.188 ± 0.825 | | 0.043 ± 0.851 | | 2.681 | 0.007 |
| -4 -56 34 | 0.463 ± 1.103 | | 0.218 ± 1.117 | | 3.419 | 0.001 |
| 10 36 -12 | -0.027 ± 0.885 | | -0.102 ± 0.926 | | 1.277 | 0.202 |
| 2 -28 64 | -0.099 ± 1.035 | | -0.099 ± 0.980 | | 0.001 | 0.999 |
| 6 44 2 | -0.124 ± 1.247 | | -0.208 ± 1.233 | | 1.042 | 0.298 |

All values are mean ± SD. *two-sample t test.

**Supplementary Table S4.** Sex differences in the correlations between β estimate (social – random) of the ROIs (Figure 3A, B, and C) and “AR_SOC_ – AR_RAN_”.

| β estimate  (social – random) | Men (n=458) | | Women (n=501) | | Slope test* | |
| --- | --- | --- | --- | --- | --- | --- |
|  | r | p | r | p | Z | p |
| *All - positive* |  |  |  |  |  |  |
| 0 -18 64 | 0.136 | 0.004 | 0.182 | 0.000 | -0.73 | 0.4654 |
| *All - negative* |  |  |  |  |  |  |
| All clusters | -0.285 | 0.000 | -0.144 | 0.001 | -2.28 | 0.0226 |
| -30 48 12 | -0.214 | 0.000 | -0.117 | 0.009 | -1.54 | 0.1236 |
| -42 18 2 | -0.238 | 0.000 | -0.095 | 0.034 | -2.27 | 0.0232 |
| -44 16 40 | -0.193 | 0.000 | -0.121 | 0.007 | -1.14 | 0.2543 |
| -4 14 50 | -0.245 | 0.000 | -0.150 | 0.000 | -1.53 | 0.126 |
| 38 24 -2 | -0.276 | 0.000 | -0.126 | 0.005 | -2.42 | 0.0155 |
| *Men* |  |  |  |  |  |  |
| All clusters | -0.308 | 0.000 | -0.085 | 0.058 | -3.59 | 0.0003 |
| -24 -38 -16 | -0.237 | 0.000 | 0.007 | 0.876 | -3.83 | 0.0001 |
| -30 46 12 | -0.231 | 0.000 | -0.027 | 0.551 | -3.21 | 0.0013 |
| -34 -62 -30 | -0.213 | 0.000 | -0.081 | 0.072 | -2.08 | 0.0375 |
| -44 18 0 | -0.244 | 0.000 | -0.064 | 0.152 | -2.85 | 0.0044 |
| -46 18 40 | -0.235 | 0.000 | -0.060 | 0.181 | -2.77 | 0.0056 |
| 12 24 28 | -0.264 | 0.000 | -0.114 | 0.011 | -2.4 | 0.0164 |
| 38 26 -2 | -0.285 | 0.000 | -0.089 | 0.047 | -3.14 | 0.0017 |
| *Women* |  |  |  |  |  |  |
| All clusters | 0.036 | 0.442 | 0.227 | 0.000 | -3.83 | 0.0001 |
| -4 -56 34 | -0.009 | 0.851 | 0.214 | 0.000 | -3.49 | 0.0005 |
| 10 36 -12 | 0.109 | 0.020 | 0.249 | 0.000 | -2.23 | 0.0257 |
| 2 -28 64 | 0.093 | 0.047 | 0.209 | 0.000 | -1.83 | 0.0673 |
| 6 44 2 | -0.014 | 0.764 | 0.228 | 0.000 | -3.79 | 0.0002 |

*Slope test to examine sex differences in the correlation. Two-tailed p values are shown. “All clusters”: all clusters combined; individual clusters are identified by their MNI coordinates.

**Supplementary Figure S1.**

**
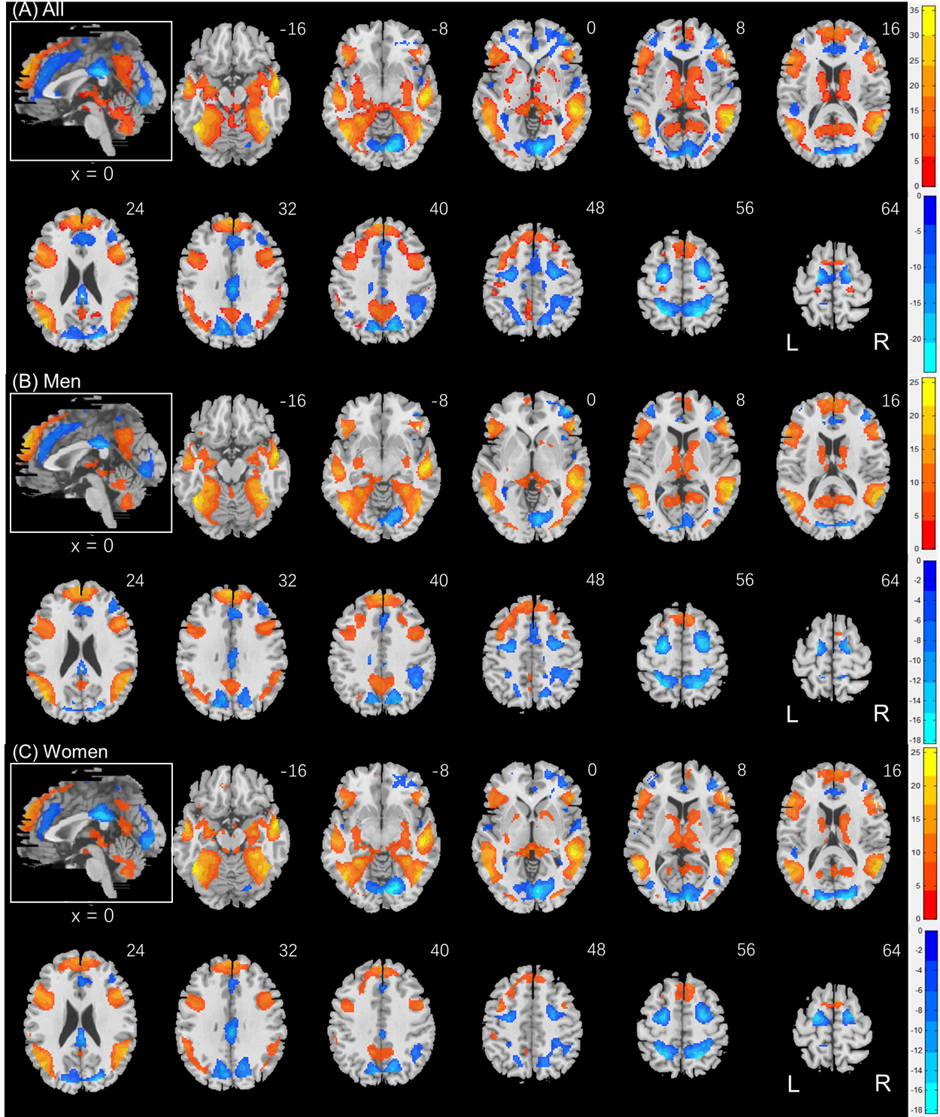
**

**Supplementary Figure S1.** One-sample t test of social vs. random on **(A)** the entire cohort, **(B)** men, and **(C)** women. Voxel p<0.05, FWE corrected. Color bars showed voxel T values. Warm color: social > random; cool color: random > social. Neurological orientation: right = right.
